# The Complete Chloroplast Genomes of *Bulbophyllum* (Orchidaceae) Species: Insight into Genome Structure Divergence and Phylogenetic Analysis

**DOI:** 10.3390/ijms25052665

**Published:** 2024-02-25

**Authors:** Yuwei Wu, Meng-Yao Zeng, Huan-Xin Wang, Siren Lan, Zhong-Jian Liu, Shibao Zhang, Ming-He Li, Yunxiao Guan

**Affiliations:** 1 Key Laboratory of National Forestry and Grassland Administration for Orchid Conservation and Utilization at Landscape Architecture and Arts, Fujian Agriculture and Forestry University, Fuzhou 350002, China; 1211775050@fafu.edu.cn (Y.W.); zmy13860927342@163.com (M.-Y.Z.); 3211726052@fafu.edu.cn (H.-X.W.); lkzx@fafu.edu.cn (S.L.); zjliu@fafu.edu.cn (Z.-J.L.); 2 Fujian Colleges and Universities Engineering Research Institute of Conservation and Utilization of Natural Bioresources, Fujian Agriculture and Forestry University, Fuzhou 350002, China; 3 Key Laboratory of Economic Plants and Biotechnology, Kunming Institute of Botany, Chinese Academy of Sciences, Kunming 650201, China; sbzhang@mail.kib.ac.cn

**Keywords:** *Bulbophyllum*, chloroplast genome, molecular markers, phylogenetics analysis

## Abstract

*Bulbophyllum* is one of the largest genera and presents some of the most intricate taxonomic problems in the family Orchidaceae, including species of ornamental and medical importance. The lack of knowledge regarding the characterization of *Bulbophyllum* chloroplast (cp) genomes has imposed current limitations on our study. Here, we report the complete cp genomes of seven *Bulbophyllum* species, including *B*. *ambrosia*, *B*. *crassipes*, *B*. *farreri*, *B*. *hamatum*, *B*. *shanicum*, *B*. *triste*, and *B*. *violaceolabellum*, and compared with related taxa to provide a better understanding of their genomic information on taxonomy and phylogeny. A total of 28 *Bulbophyllum* cp genomes exhibit typical quadripartite structures with lengths ranging from 145,092 bp to 165,812 bp and a GC content of 36.60% to 38.04%. Each genome contained 125–132 genes, encompassing 74–86 protein-coding genes, 38 tRNA genes, and eight rRNA genes. The genome arrangements, gene contents, and length were similar, with differences observed in *ndh* gene composition. It is worth noting that there were exogenous fragment insertions in the IR regions of *B*. *crassipes*. A total of 18–49 long repeats and 38–80 simple sequence repeats (SSRs) were detected and the single nucleotide (A/T) was dominant in *Bulbophyllum* cp genomes, with an obvious A/T preference. An analysis of relative synonymous codon usage (RSCU) revealed that leucine (Leu) was the most frequently used codon, while cysteine (Cys) was the least used. Six highly variable regions (*rpl32*-*trnL^UAG^* > *trnT^UGU^*-*trnL^UAA^* > *trnF^GAA^*-*ndhJ* > *rps15*-*ycf1* > *rbcL*-*accD* > *psbI*-*trnS^GCU^*) and five coding sequences (*ycf1* > *rps12* > *matK* > *psbK* > *rps15*) were identified as potential DNA markers based on nucleotide diversity. Additionally, 31,641 molecular diagnostic characters (MDCs) were identified in complete cp genomes. A phylogenetic analysis based on the complete cp genome sequences and 68 protein-coding genes strongly supported that 28 *Bulbophyllum* species can be divided into four branches, sects. *Brachyantha*, *Cirrhopetalum*, and *Leopardinae*, defined by morphology, were non-monophyly. Our results enriched the genetic resources of *Bulbophyllum*, providing valuable information to illustrate the complicated taxonomy, phylogeny, and evolution process of the genus.

## 1. Introduction

*Bulbophyllum*, comprising approximately 2200 species, is one of the largest genera of Orchidaceae and serves as an excellent model system for investigating orchid biodiversity [1,2,3]. Its distribution spans pantropical regions, including Africa, Madagascar, the Americas, and the Asia–Pacific region [4]. Members of this genus exhibit epiphytic or lithophytic habits and typically possess one- or two-leaved pseudobulbs with a labellum attached to the base of the floral column via an elastic hinge [4,5]. *Bulbophyllum* demonstrates remarkable adaptability, flourishing in a variety of environments including subtropical dry forests and wet montane cloud forests [4,6]. Owing to its morphologically diverse lateral sepals that vary in size, shape, color, and surface ornamentation, *Bulbophyllum* has economic importance that is attributable to its ornamental uses [5]. Additionally, the aromatic compounds found in these orchids are significant for their medical benefits [7,8].

*Bulbophyllum* has a complex taxonomic history, with numerous proposals for generic delimitations and infrageneric classifications based on morphological characters since its establishment by Thouars in 1822 [9,10,11,12,13,14]. Two main perspectives exist on the morphological division of *Bulbophyllum*: either dividing the genus into multiple sections or categorizing the broad genus into several genera. Statistically, more than 50 genera have been merged into *Bulbophyllum* (e.g., *Cirrhopetalum* Lindl., *Drymoda* Lindl., *Monomeria* Lindl., *Trias* Lindl., and *Sunipia* Lindl.), and approximately 70–80 sections have been proposed alone in the Asia–Pacific region [4,15,16].

Phylogenetic analyses of *Bulbophyllum* using DNA sequence data have made significant progress recently. Most phylogenetic results supported the monophyly of a broadly defined *Bulbophyllum* and its continental taxa, such as Asian, African, and Neotropical clades [1,4,17]. Hu et al. reconstructed the phylogenetic relationship in the Asian *Cirrhopetalum* alliance of *Bulbophyllum* based on combining four DNA sequence data (nrDNA: ITS, *Xdh*; cp DNA: *matK* and *psbA*-*trnH*, 117 taxa), supporting an amended *Cirrhopetalum* alliance was monophyly [5]. Based on eight DNA sequence data (nrDNA: ITS, *Xdh*, *OrcPI*; cp DNA: *atpI*-*atpH*, *ycf1*, *matK*, *trnD*-*trnE*, *psbA*-*trnH*, 179 taxa), Gamisch et al. divided the Malagasy taxa into four clades [18]. These studies have clarified the phylogenetic relationships of different regions in this group, but nodal support values of the main clades or lineages were moderate to low or lacking for some relationships. The number of accepted species continues to grow as new discoveries are reported [19,20,21]; taxonomic work on *Bulbophyllum* became a major challenge, and further investigation into the relationships within the genus necessitates more detailed study.

With the continuous reduction in sequencing costs, the chloroplast (cp) genome has become a pivotal tool for investigating phylogenetic relationships within complex taxa. The cp genome offers several advantages, including a unique mode of inheritance, highly conserved genome structure, and a moderate evolutionary rate [22,23]. Owing to these unique characteristics, cp genomes are widely used to explore the phylogenetic relationship among orchid clades. Liu et al. reconstructed the phylogenetic relationships of the *Cleisostoma*–*Gastrochilus* clades in Aeridinae based on the cp genomes, robustly supporting that this clade can be divided into six subclades with higher support rates and more stable topological structures than before [24]. Additionally, many studies have comprehensively compared differences in orchid cp genomes to understand the structural characteristics and evolution patterns, such as *Pholidota* (13 species) and *Paraphalaenopsis* (three species) [25,26]. Yang et al. compared and analyzed cp genomes of 18 species from Asian and Neotropical *Bulbophyllum*. The results show that the cp genome structure of Asian and Neotropical clades was different due to selection pressures under the condition of geographical isolation [27]. Furthermore, integrative analyses of multiple cp genomes can help to develop applicable molecular markers for species identification [28]. Five highly variable regions (*ycf1*, *ndhA*, *ndhF*, *trnQ*, and *trnK*), the potential DNA markers, were found in four *Liparis* cp genomes [29]. Tang et al. analyzed the cp genomes of sect. *Macrocaulia* in *Bulbophyllum* and proposed 20 intergenic regions and three coding genes of the most variable hotspot regions as candidate effective molecular markers [30]. Although the species identification rate of multiple DNA molecular markers has been improved to different degrees, the species identification rate of closely related groups is still not optimistic [31]. Complete cp genome sequences harbor many more sequence variations, making them far more sensitive and effective than standard DNA barcodes, referred to as ultrabarcodes, can effectively improve the identification rate [32]. Sawicki et al. analyzed the cp genomes of *Apopellia endiviifolia* using the ultrabarcode technique. They suggested that the genomes could be clearly separated into two evolutionary lineages, and the number of detected molecular diagnostic characters (MDCs) indicated a level of genetic divergence in the dataset of cp genomes [33].

To date, only a few cp genomes of *Bulbophyllum* have been sequenced. Detailed cp genomic comparisons and phylogenetic analyses are lacking, which hinders our ability to further elucidate its interspecific relationships. In order to further clarify the phylogenetic relationships among species of the genus and to obtain useful genetic resources, we sequenced and assembled the cp genomes of seven *Bulbophyllum* species (*B*. *ambrosia*, *B*. *crassipes*, *B*. *farreri*, *B*. *hamatum*, *B*. *shanicum*, *B*. *triste*, *B*. *violaceolabellum*) and compared them with other *Bulbophyllum* species published to investigate their relationships. Our results will provide valuable information for cp genome evolution, phylogenetic relationships, and species identification of Orchidaceae.

## 2. Results

### 2.1. General Characteristics of the Chloroplast Genomes

**Figure 1:**
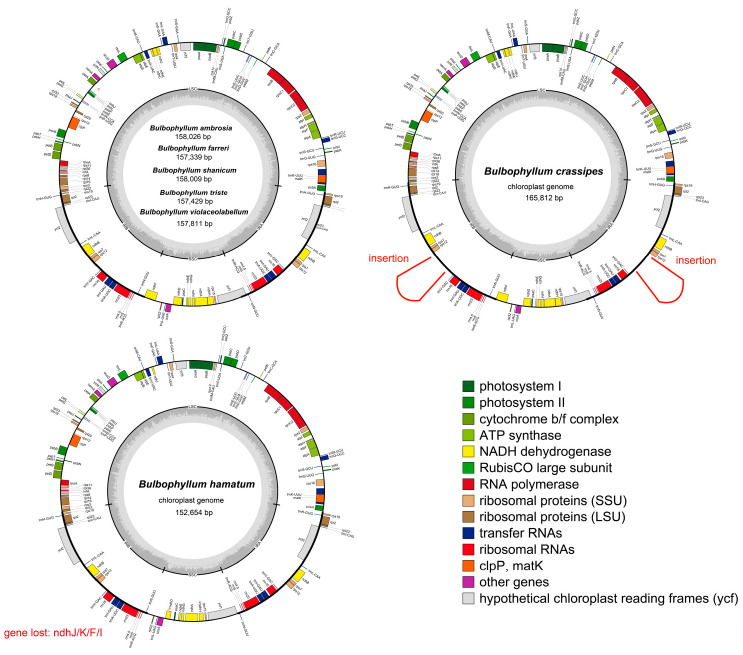
Chloroplast genome maps for seven *Bulbophyllum* species. Genes on the inside of the large circle are transcribed clockwise, and those on the outside are transcribed counter-clockwise. The color coding of the genes is determined according to their annotation functions. The GC content of the chloroplast genomes is represented by the dashed area. Pseudogenes are marked by Ψ.

The seven newly sequenced *Bulbophyllum* cp genomes were circular with the typical quadripartite structure, including a large single copy (LSC), a small single copy (SSC), and a pair of inverted repeats (IRs) (Figure 1). We combined the published cp genomes of 21 *Bulbophyllum* orchids with this study’s seven species to compare the basic cp genome features within the genus. The number of genes, GC content, etc., of the 28 cp genomes are summarized in Table 1. As shown in Table 1, the 28 *Bulbophyllum* cp genome sizes ranged from 145,092 bp (*B*. *kwangtungense*) to 165,812 bp (*B*. *crassipes*). The cp genomes were variable in LSC and SSC regions, with 77,088 to 87,177 bp and 11,089 to 18,632 bp, while being conserved in IR regions, with sizes ranging from 25,465 to 30,927 bp. The GC content was relatively consistent, ranging from 36.60% to 38.04%, and the distribution of the GC content across different regions was uneven, with about 43.18%, 34.93%, and 29.67% for the IR, LSC, and SSC regions, respectively (Table 1).

Each cp genome was annotated with a total of 106-113 unique genes, which included 68-79 protein-coding genes, 30 transfer RNAs (tRNAs), and four ribosomal RNAs (rRNAs) (Table 1). Most genes existed as single copies in either LSC or SSC regions. However, 19 genes were duplicated in IRs, encompassing seven protein-coding genes (*ycf1*, *rpl2*, *rps7*, *ndhB*, *ycf2*, *rpl23*, and *rps12*), eight tRNAs (*trnN^GUU^*, *trnR^ACG^*, *trnA^UGC^*, *trnL^GAU^*, *trnV^GAC^*, *trnL^CAA^*, *trnI^CAU^*, and *trnH^GUG^*), and four rRNAs (*rrn23*, *rrn16*, *rrn5*, and *rrn4.5*). Nine protein-coding genes and six tRNA genes contained one intron each, whereas genes *ycf3* and *clpP* possessed two introns. The lengths of introns varied among different genes, with the longest intron found in the *trnK^UUU^* gene. Notably, the *ndh* genes were truncated or completely lost in more than half of the species (Table 1, Supplementary Table S10). The highest degree of loss was the *ndhF* gene, which was observed in 11 species. The highest degree of pseudogenization was the *ndhD* gene, which was pseudogenized in nine species. The species with the simultaneous pseudogenization and loss of the *ndh* gene are *B*. *disciflorum*, *B*. *exaltatum*, *B*. *granulosum*, *B*. *hamatum*, *B*. *inconspicuum*, *B*. *kwangtungense*, *B*. *mentosum*, *B*. *ningboense*, *B*. *pingnanense*, *B*. *plumosum*, and *B*. *tianguii*. All functional genes could be categorized into three groups: those related to self-replication, photosynthesis, and others (Supplementary Table S1).

### 2.2. Repeat Sequence Characterization

**Figure 2:**
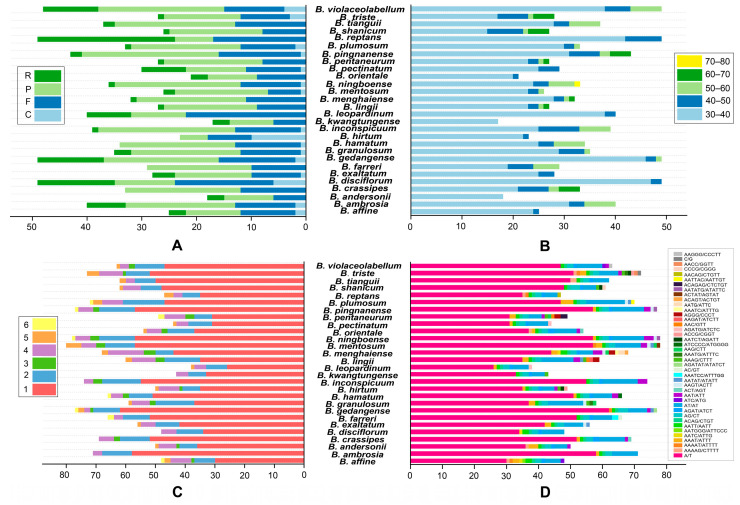
Summary of sequence repeats across the 28 *Bulbophyllum* cp genomes. (**A**) Variation in repeat abundance and type; (**B**) number of long repeats by sequence length; (**C**) frequency of identified SSR motifs (mono-, di-, tri-, tetra-, penta-, and hexa-); (**D**) frequency of classified repeat types (considering sequence complement).

We identified four types of long repeats—palindromic (P), forward (F), complementary (C), and reverse (R) elements (Figure 2A, Supplementary Table S2) in 28 *Bulbophyllum* cp genomes. Among these, all four categories were observed in 14 species, while 12 species contained three categories of repeats (C/R, F, and P); two species (*B*. *crassipes* and *B*. *farreri*) exhibited two categories (P and F). The number of long-repeat sequences ranged from 17 (*B*. *kwangtungense*) to 49 (*B. disciflorum*, *B*. *gedangense*, *B*. *reptans*, and *B*. *violaceolabellum*). Across these 28 cp genomes, P was the most prevalent, ranging from five occurrences in *B*. *hirtum* to 25 occurrences in *B*. *inconspicuum* and *B*. *pingnanense*. The *Bulbophyllum* cp genomes had fewer R and C repeats, and the highest counts of the two types were 25 Rs in *B*. *reptans* and 10 Cs in *B*. *hirtum*, respectively. Long-repeat sequences in the range of 30–40 bp were the most frequently observed and ranged from 15 occurrences in *B*. *shanicum* to 47 occurrences in *B*. *disciflorum*. *B*. *inconspicuum* displayed the highest count of 40–50 bp repeats. The 50–60 bp repeat sequences were detected in 18 *Bulbophyllum* species, ranging from one to six occurrences. The 60–70 bp repeat sequences were only present in *B*. *crassipes*, *B*. *lingii*, *B*. *menghaiense*, *B*. *pentaneurum*, *B*. *pingnanense*, *B*. *shanicum*, and *B*. *triste*, ranging from one to four occurrences. The longest repeat sequences were 77 bp in *B. ningboense* (Figure 2B, Supplementary Table S3).

A total of 38 (*B*. *leopardinum*) to 80 (*B*. *mentosum*) SSRs were detected in the cp genome of the 28 *Bulbophyllum* species, and six categories of SSRs (mono-, di-, tri-, tetra-, penta-, and hexanucleotide repeats) were identified (Figure 2C,D and Supplementary Tables S4 and S5). And SSRs were mostly located in the intergenic region of LSC (Supplementary Figure S1). Mono-nucleotide repeats (SSR loci A/T) were the most abundant, accounting for 58.3% (*B*. *lingii*) to 81.7% (*B*. *ambrosia*), with counts varying from 47 to 58. This was followed by di-nucleotide repeats (6 to 13 occurrences, 8.8.% to 22.0%), tri-nucleotide repeats (zero to four occurrences, 5.9%), tetra-nucleotide repeats (2 to 13 occurrences, 2.6% to 17.6%), penta-nucleotide repeats (zero to five occurrences, 6.4%), and hexa-nucleotide repeats, with the least number of SSRs (zero to two occurrences, 4.1%). All mononucleotide SSRs belonged to the A or T type, and the majority of di-, tri-, tetra-, penta-, and hexa-nucleotide SSRs were particularly rich in A or T (Figure 2D, Supplementary Table S5). In general, the distribution pattern of SSRs was uneven across the 28 species. The mono-, di-, and tetra- nucleotide repeat categories were observed in all species, while tri- and penta-nucleotide repeats were absent in 10 different species. Hexa-nucleotide repeats were only present in *B*. *affine*, *B*. *farreri*, *B*. *gedangense*, *B*. *hamatum*, *B*. *ningboense*, *B*. *pentaneurum*, *B*. *pingnanense*, and *B*. *plumosum*.

### 2.3. Relative Synonymous Codon Usage Analysis

**Figure 3:**
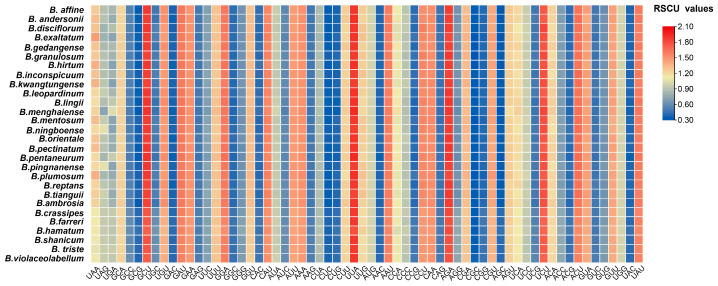
RSCU values of the codons in the 28 *Bulbophyllum* cp genomes.

We analyzed a total of 68 protein-coding genes among the 28 *Bulbophyllum* cp genomes, with the exception of the *ndh* genes due to incomplete gene loss and pseudogenization. These genes were encoded by a range of 17,226 codons in *B*. *plumosum* to 22,758 codons in *B*. *shanicum* (Figure 3, Supplementary Table S6). The codon usage patterns revealed a highly conserved codon usage bias (CUB). Leucine (Leu) was one of the most frequently occurring amino acids, appearing a total of 57,130 times across all 28 cp genomes. In contrast, cysteine (Cys) was the least frequent, occurring only 6515 times. An analysis of the relative synonymous codon usage (RSCU) indicated that UUA and AGA had the highest CUB, with average values of 1.934 and 1.894, respectively, while CGC and CUC had the lowest CUB, with average values of 0.374 and 0.397, respectively. Among the three stop codons, the frequency of UAA was the highest, accounting for 39.9%. The results also showed that 30 codons exhibited RSCU values greater than one, and 32 codons exhibited values less than one (Figure 3, Supplementary Table S6). The RSCU values of AUG encoding for methionine (Met) and UGG encoding for tryptophan (Trp) were determined to be one in all seven species.

### 2.4. Expansion and Contraction of IRs, Sequence Divergence, and Nucleotide Diversity

**Figure 4:**
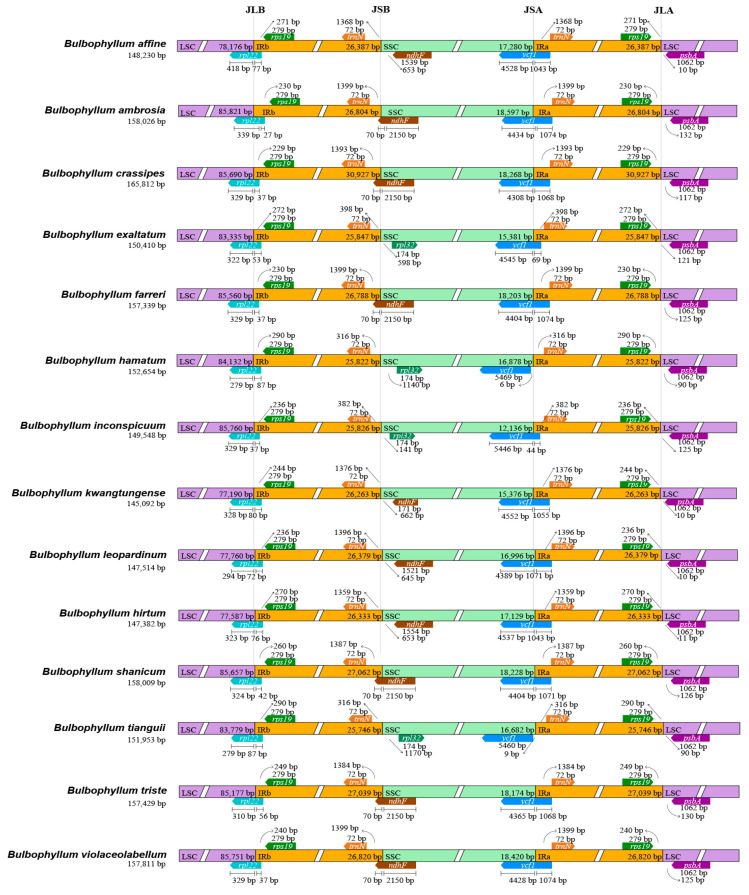
Comparison of junctions between the LSC, SSC, and IR regions among 14 *Bulbophyllum* cp genomes. JLB, JSB, JSA, and JLA denoted the junction sites of LSC/IRb, IRb/SSC, SSC/IRa, and IRa/LSC, respectively.

A comprehensive comparison of the boundaries between the LSC, IRs, and SSC regions was conducted across the 28 *Bulbophyllum* species (Figure 4, Supplementary Figure S2). The junctions between the IRs and SC regions exhibited a high degree of conservation. In the cp genomes of these 28 *Bulbophyllum* species, several key genes, namely *rpl22*, *ndhF*, *ycf1*, *rps19*, and *psbA*, were found at the junction of the LSC/IRb, IRb/SSC, SSC/IRa, and IRa/LSC borders. The *rpl22* gene, spanning from LSC to IRb, was primarily located in the LSC region and ranged from 279 to 423 bp in length. *B*. *hamatum* and *B*. *tianguii*, comprising 279 bp in the LSC region and 87 bp in the IRb region, were the shortest. Furthermore, the IRb/SSC border of *B*. *ambrosia*, *B*. *crassipes*, *B*. *farreri*, *B*. *gedangense*, and *B*. *mentosum* was located in the *ndhF* pseudogene, with just 70 bp located in the IRb region. The *ndhF* pseudogene was close, too, but did not overlap with the IRb/SSC junction in *B*. *affine*, *B*. *andersonii*, *B*. *disciflorum*, *B*. *hirtum*, *B*. *kwangtungense*, *B*. *leopardimum*, *B*. *orientale*, *B*. *pectinatum*, or *B*. *reptans*. Within the SSC/IRa (JSA) region, the *ycf1* gene spanned the SSC/IRa boundary, primarily residing in the SSC region, with lengths ranging from 4308 bp (*B*. *crassipes*) to 5469 bp (*B*. *hamatum*). In the case of *B*. *hamatum* and *B*. *tianguii*, the *ycf1* gene was positioned to the left side of the JSA line, with a distance of 6 bp and 9 bp, respectively. In the IRa/LSC (JLA) region, the *rps19* gene was situated on the left side of the JLA line, and the distance from *rps19* to the JLA line ranged from 229 bp (*B*. *crassipes*) to 290 bp (*B*. *hamatum*). The *psbA* gene was located on the right side of the JLA line, with distances ranging from 10 bp (*B*. *kwangtungense*, *B*. *leopardinum*, and *B*. *pectinatum*) to 132 bp (*B*. *ambrosia*) (Figure 4, Supplementary Figure S2).

The divergence of sequences in the cp genomes of 28 *Bulbophyllum* species was plotted using the mVISTA program with the annotated *B*. *affine* (LC556091) sequence as a reference (Supplementary Figure S3). The results revealed sequences with significant conservation in *Bulbophyllum* cp genomes, particularly in the coding region. The highest variation was observed in the SSC region, followed by the LSC region and IR regions. Mauve visualization graphs also indicated that no significant gene rearrangement was detected among these cp genomes (Supplementary Figure S4). Exogenous fragment insertions were detected at *rps12*~*trnV^GAG^* in the IR regions of *B*. *crassipes*, with a length of 3975 bp (Figure 1).

**Figure 5:**
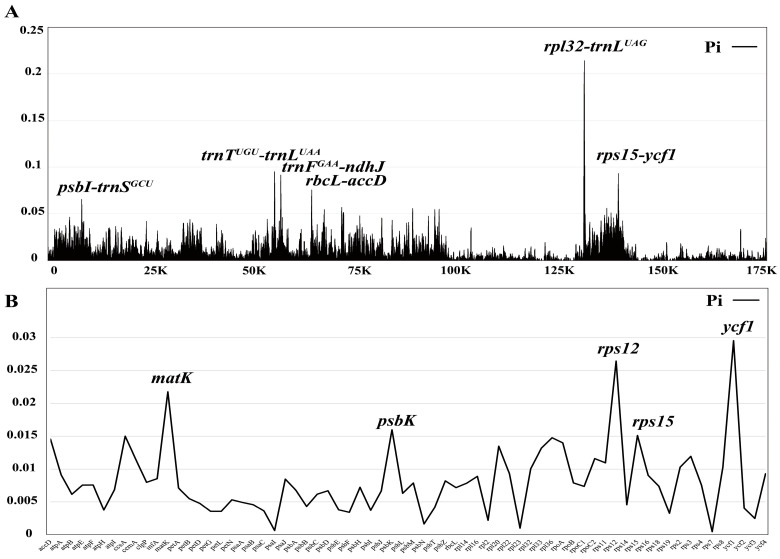
The nucleotide diversity (Pi) of 28 *Bulbophyllum* cp genomes and 68 protein-coding sequences. (**A**) For the nucleotide diversity of the complete cp genomes using a sliding window test, four mutation hotspot regions were annotated. The window size was set to 100 bp and the sliding window size was 25 bp. X-axis, the position of the midpoint of a window; Y-axis, Pi values of each window. (**B**) The nucleotide diversity of 68 protein-coding sequences. X-axis, gene; Y-axis, Pi values of each gene.

The nucleotide diversity value (Pi) for the coding regions and intergenic regions was calculated using DnaSP to further analyze the mutation hotspots in 28 *Bulbophyllum* species. The results showed that the Pi values ranged from 0 to 0.21413 (*rpl32*-*trnL^UAG^*) (Figure 5A, Supplementary Table S7). The IR regions exhibited the highest conservation, with a value of 0.0035. The SSC region displayed the greatest nucleotide diversity (Pi = 0.0307), followed by the LSC region (0.0141). According to the ranking of the Pi values, six hypervariable regions were identified for candidate barcodes, including *rpl32*-*trnL^UAG^* (0.21413), *trnT^UGU^*-*trnL^UAA^* (0.09500), *trnF^GAA^*-*ndhJ* (0.09138), *rps15*-*ycf1* (0.09315), *rbcL*-*accD* (0.07534), and *psbI*-*trnS^GCU^* (0.06529). Additionally, the protein-coding genes displayed higher conservation (Figure 5B, Supplementary Table S8). Among these genes, *ycf1* (0.02956), *rps12* (0.02643), *matK* (0.02178), *psbK* (0.01599), and *rps15* (0.01511) exhibited the highest Pi values appropriate for phylogeny.

### 2.5. Molecular Diagnostic Characters (MDCs)

The ABGD analysis revealed a consistent count of species division; there were 20 groups in the 28 *Bulbophyllum* species. The prior intraspecific distance ranged between 0.0021 and 0.0010. The results correctly recognized 14 species, and the other 14 sequences were ambiguous (*B*. *andersonii* with *B*. *kwangtungense*; *B*. *crassipes* with *B*. *orientale*; *B*. *hamatum* with *B*. *tianguii* and *B*. *violaceolabellum*; *B*. *inconspicuum* with *B*. *ningboense* and *B*. *pingnanense*; *B*. *leopardinum* with *B*. *pectinatum*; and *B*. *shanicum* with *B*. *triste*) (Supplementary Table S11).

**Figure 6:**
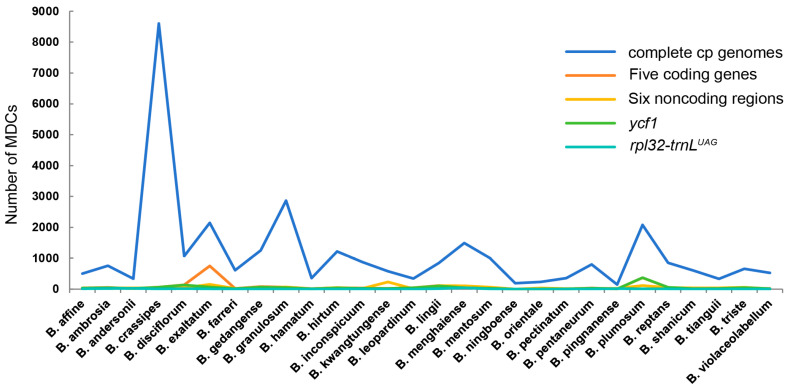
Molecular diagnostic characteristics (MDCs) in whole cp genomes and four datasets of 28 *Bulbophyllum* species.

A complete cp genome analysis revealed 31,641 MDCs in the 28 *Bulbophyllum* species, which comprised 17.73% of the total length (Figure 6, Supplementary Table S12). The number of MDCs that distinguished species from the others of the genus was extremely variable, ranging from 149 to 8603 (*B*. *crassipes*). The MDC analysis was also carried out using the four datasets (five coding genes, six noncoding regions, *ycf1*, and *rpl32*-*trnL^UAG^*) obtained by Pi values. The higher number of MDCs identified was with the dataset of five coding genes; the sum of MDCs was 2203 (26.32% of the total length), ranging from 3 to 750 (*B*. *exaltatum*). The sums of MDCs of the latter three datasets were 1534, 1435, and 249, accounting for 33.48%, 24.08%, and 29.05%, respectively. The datasets of *ycf1* and six noncoding regions ranged from 2 to 372 (*B*. *plumosum*) and 3 to 232 (*B*. *kwangtungense*), respectively. The lowest number of MDCs was identified in the dataset of *rpl32*-*trnL^UAG^*; the number of MDCs was 45, and three species did not have MDCs.

### 2.6. Phylogenetic Analysis

**Figure 7:**
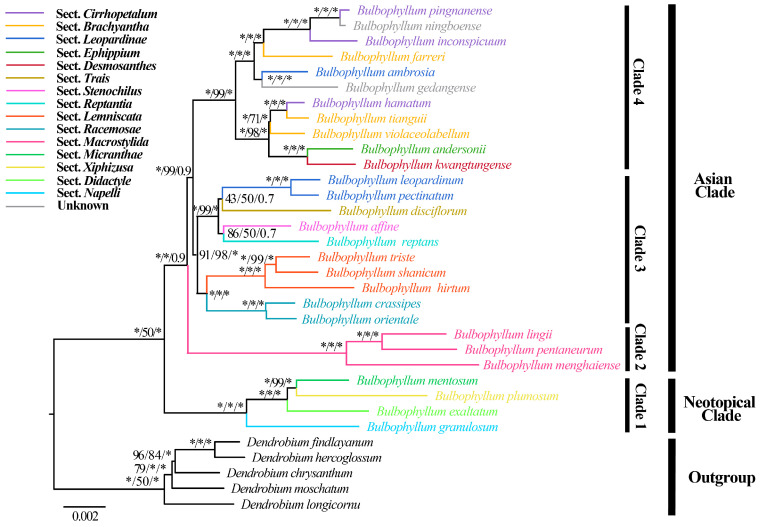
Phylogenetic tree of 28 *Bulbophyllum* species and five outgroups based on complete cp genomes. The numbers near the nodes are bootstrap percentages and Bayesian posterior probabilities (BP_ML_, BP_MP_, PP). * Node is 100 bootstrap percentage or 1.00 posterior probability. The annotations of sections referred to Pridgeon et al. [4] and Hu et al. [5] are highlighted by the color of branches.

The phylogenetic analysis of 28 *Bulbophyllum* species, based on two datasets comprising complete cp genomes and 68 protein-coding genes, revealed that the species formed four major clades (Figure 7, Supplementary Figure S5). The alignment matrix of complete cp genomes was 131,138 bp, with 12,031 variable sites and 6201 parsimony informative sites. The matrix of 68 protein-coding genes was 59,417 bp and included 4869 variable sites, along with 2404 parsimony informative sites. The topologies remained largely consistent within the two datasets, demonstrating strong support based on complete cp genomes (Bootstrap Support, BS ≥ 98; posterior probability, PP = 1.00), while the support was relatively moderate, inferred by 68 protein-coding genes (BS ≥ 75, PP ≥ 0.70) (Figure 7, Supplementary Figure S5). Clade 1 (Neotropical clade) consisted of four species from different sections and Clade 2 primarily consisted of species from sect *Macrocaulia* with robust support. In Clade 3, sects *Lemniscata* (*B. shanicum*, *B. triste*, and *B. hirtum*) and *Racemosae* (including *B. crassipes* and *B*. *orientale*) were sister groups with generally high support values in one subclade, while another subclade consisted of species from sects *Leopardinae*, *Trias*, *Stenochilus*, and *Repantia*. Clade 4 contained species assigned to sects *Cirrhopetalum*, *Brachyantha*, *Leopardinae*, *Ephippium*, and *Desmosanthes*. A single species of sect *Brachyantha* (*B. farreri*) appeared as a sister to two species from sect *Cirrhopetalum* (including *B*. *pingnanense* and *B*. *inconspicuum*) with strong support (BP = 100, PP = 1.00). Additionally, *B. ambrosia* and *B. gedangense* formed a separate and strongly supported clade. Notably, *B. hamatum*, a newly described species belonging to sect *Cirrhopetalum* and recently published by Yan et al. [19], appeared as a sister to *B. tianguii* (sect *Brachyantha*). Subsequently, *B. violaceolabellum* (sect *Brachyantha*) was also a sister to these two species with strong support (BP = 100, PP = 1.00).

## 3. Discussion

### 3.1. The Characteristics of Chloroplast Genomes

Owing to the highly conserved structure, uniparental inheritance, and mutation rates between those shown in the mitochondrial and nuclear genomes, cp genomes have been widely employed for investigating phylogenetic relationships [22,23]. Recently, orchids have become a focal point in phylogenetic studies due to their rich diversity, wide distribution, and epiphytic habits. With the decreasing costs of sequencing, an increasing number of cp genome evolutions in Orchidaceae have been studied [34,35]. The genus *Bulbophyllum* serves as one of the representative groups of orchid biodiversity [1,2,4]; the cp genomes of their diversity patterns and evolutionary adaptations attract much attention [24,30,36].

This study sequenced the complete cp genomes of seven orchid species in the genus *Bulbophyllum* and compared them with other 21 *Bulbophyllum* species in order to broaden the knowledge about the genome organization and molecular evolution of the Orchidaceae species. The obtained seven cp genomes of the *Bulbophyllum* species in this study possessed a typical quadripartite structure, with the genome sizes of these cp genomes varying from 145,092 bp (*B*. *kwangtungense*) to 165,812 bp (*B*. *crassipes*), and the GC content ranging from 36.60% (*B*. *plumosum*) to 38.04% (*B*. *leopardinum*), all of which fell within the normal range of cp genomes reported in previous studies [37,38]. The gene order and content were not different from those of other closely related *Bulbophyllum* species [27,30,36,39,40]. Notably, an exogenous fragment insertion appeared at *rps12*~*trnV^GAG^* in the IR regions of *B*. *crassipes*, leading to the length of the IRs being obviously larger than other *Bulbophyllum* species (Figure 1 and Figure 4, Supplementary Figure S2). The source remains to be further explored.

Although the general structure of *Bulbophyllum* cp genomes is conserved, differences in *ndh* gene composition were detected. The *ndh* genes encode the thylakoid NADH complex [41], which is frequently pseudogenized or lost in Orchidaceae [42,43]. Recently, studies of the orchid cp genomes have revealed that rampant independent loss of the *ndh* genes occurred in different orchid clades. The cp genome of *E*. *pusilla* contains truncated versions of *ndhJ*, *C*, *D*, *B*, *G*, and *H*, and lacks sequences for *ndhK*, *F*, *E*, *A*, and *I* [44], and the pseudogenization of *ndh* genes in the *Cleisostoma*-*Gastrochilus* clades is widespread [24]. In this study, all of the cp genomes showed evidence of gene pseudogenization or loss except *B*. *affine*, *B*. *andersonii*, *B*. *crassipes*, *B*. *farreri*, *B*. *gedangense*, *B*. *hirtum*, *B*. *leopardinum*, *B*. *lingii*, *B*. *menghaiense*, *B*. *orientale*, *B*. *pectinatum*, *B*. *pentaneurum*, *B*. *shanicum*, *B*. *triste*, and B. *violaceolabellum* (Table 1, Supplementary Table S10). Some studies have suggested that the inactivation of *ndh* genes may be associated with epiphytic habitats [45] and connected to the extreme water availability and use of CAM (Crassulacean acid metabolism) photosynthesis [24,46], such that the *ndh* genes were extensively pseudogenized in *Cymbidium mannii*, an epiphyte with constitutive CAM [47]. Although *Bulbophyllum* is primarily an epiphytic group and utilizes the CAM pathway [18,48], more research is needed to understand the relationship between the evolution of the CAM pathway or growth form and the cp genomes.

### 3.2. Repeat Sequence Analysis

As an inherent variation, long-repeat sequences with lengths greater than 30 bp are universal in angiosperms and considered to play an important role in genome stability and structural variation [49]. There were abundant long-repeat sequences in the cp genomes of *Bulbophyllum* species in previous studies [36], and a total of 18–49 long repeats were detected in our study (Figure 2B). The palindromic (P) and forward (F) repeats were the most common long-repeat sequences in our study (Figure 2A). Slight variation in the number of repeat units and their proportions occurred in different species. Additionally, the GC content of IR regions was much higher than that of the LSC and SSC regions (Table 1), and these characteristics were also revealed in other plant species, primarily because of the presence of rRNA (*rrn4.5*, *rrn5*, *rrn16*, and *rrn23*) genes in this region [50].

Simple sequence repeats (SSRs) are highly abundant and randomly distributed throughout the genome, making them valuable genetic molecular markers for population genetic relationships and phylogenetic studies [51]. A number of SSR markers were discovered in several orchid genera such as *Vanda* [52] and *Dendrobium* [53]. The most abundant SSR type was the mononucleotide repeat, and the majority of SSRs in the *Bulbophyllum* species were composed of A/T SSRs [27,30,36]. In this study, a total of 38–80 SSRs and six types of SSRs (mono-, di-, tri-, tetra-, penta-, and hexanucleotide repeats) were detected (Figure 2C,D). A/T SSRs were found to be more abundant than G/C SSRs (G/C was only detected in *B*. *triste*) and may be due to a bias towards A/T in cp genomes [54]. Of di- to hexa-nucleotide SSRs among *Bulbophyllum* species, most SSRs were specific to each species (Figure 2D). These SSRs were distributed widely and randomly throughout the chloroplast genomes, and were usually located in the intergenic spacer (IGS) region, which is consistent with angiosperm cp genomes [30]. Most of the previous studies revealed that the richness of SSR types is various in different species, which may contribute to the genetic variations among species [55]. Notably, some of the SSRs repeats were highly specific, such as AC/GT and AAATCC/ATTTGG SSRs only being detected in the cp genomes of *B*. *farreri*, AGATAT/ATATCT SSRs only being detected in *B*. *gedangense*, and ATCCCC/ATGGGG only being detected in *B*. *hamatum*. Furthermore, *B*. *crassipes* and *B*. *orientale* (the members of sect *Racemosae*) possessed ACT/AGT SSRs; *B*. *hirtum*, *B*. *shanicum*, and *B*. *triste* (the members of sect *Lemniscata*) possessed AATCT/AGATT SSRs, which is consistent with some results of the phylogenetic analysis (Figure 2C,D). Thus, these SSRs have the potential to be specific molecular markers for establishing the molecular evolutionary history and demographic diversity of *Bulbophyllum* species. These results are significant for identifying and analyzing genetic diversity in *Bulbophyllum*.

### 3.3. Codon Usage Analysis

Codons are involved in protein translation, vital for the genetic information transfer process of an organism. Codon usage bias is a significant factor in cp genome evolution, influencing gene function expression. Organisms with close genetic relationships exhibit similar codon usage bias [56]. These studies can help to clarify evolutionary relationships and improve the efficiency of gene expression in research utilizing genetic transformation [57]. More recently, a variety of orchid cp genomes have been sequenced, allowing for the comprehensive analysis of codon preferences [58,59]. The codon usage bias in *Bulbophyllum* cp genomes showed similar patterns, as indicated by the comparative analysis of RSCU values (Figure 3, Supplementary Table S6). According to the RSCU analysis, it was found that most of the frequently used codons (RSCU > 1) ended in A or U, while the less frequently used codons (RSCU < 1) ended in C or G. Among all codons, leucine (Leu) had the highest occurrence, while cysteine (Cys) had the lowest frequency. This trend is consistent with observations in most angiosperm cp genomes [60] and further demonstrates the high conservation in these 28 *Bulbophyllum* species.

### 3.4. Expansion and Contraction of IRs, Sequence Divergence, and Nucleotide Diversity

Boundary shifts between the IR and SC regions are a common occurrence in the evolution of angiosperms and are the main factors contributing to the differences in the length and gene content of cp genomes [58]. For instance, the IR region of the cp genome of *Pelargonium* × *hortorum* was expanded extensively; its length was increased to 76 kb [61]. In general, the gene arrangement of the IR/SC boundary was highly conserved (Figure 4, Supplementary Figure S2), with some differences in the IR/SSC junction detected. In *B*. *hamatum* and *B*. *tianguii*, the *ycf1* gene was completely located within the SSC region, while in the other species, the *ycf1* gene crossed over JSA. At the junction between JSB, some species lost the *ndhF* gene. This result indicated that there was no significant expansion or contraction in the IR regions of *Bulbophyllum*. This may be one of the primary factors contributing to the high conservation of the cp genome structure.

The divergent regions as molecular markers could provide abundant valuable information for DNA barcoding and phylogenetic studies, as well as phylogenetic reconstruction research using divergent hotspots [62]. Recently, various plastid markers have been proposed for Orchidaceae. Dong et al. suggested that eleven mutational hotspot regions could be used as potential DNA barcodes, including five noncoding regions (*ndhB* intron, *ccsA*-*ndhD*, *rpl33*-*rps18*, *ndhE*-*ndhG*, and *ndhF*-*rpl32*) and six coding regions (*rps16*, *ndhC*, *rpl32*, *ndhI*, *ndhK*, and *ndhF*) [63]. We identified several prominent divergent regions in this study, including *rpl32*-*trnL^UAG^*, *trnT^UGU^*-*trnL^UAA^*, *trnF^GAA^*-*ndhJ*, *rps15*-*ycf1 rbcL*-*accD*, and *psbI*-*trnS^GCU^* (Figure 5A). These regions exhibited a nucleotide diversity greater than 0.065. The *psbI*-*trnS*, *trnF*-*ndhJ* and *trnT*-*trnL* regions have been identified or utilized in previous studies of *Bulbophyllum* [27,30,64,65], further supporting previous results. Five protein-coding genes (*ycf1*, *rps12*, *matK*, *psbK*, and *rps15*) also showed high Pi values; they are still highly conserved, with nucleotide values exceeding 0.015 (Figure 5B). Furthermore, IR regions were highly conserved and had more mutation sites compared to the coding region, which is consistent with previous studies on Orchidaceae [43,58] (Figure 4, Supplementary Figure S2).

### 3.5. Molecular Diagnostic Characters (MDCs)

Within uncharacterized groups, DNA barcodes, short DNA sequences that are present in a wide range of species, can be used to assign organisms into species. Automatic Barcode Gap Discovery (ABGD) can assign these sequences into potential species based on the barcode gap [66]. A previous ABGD analysis showed that clear taxa assignments mostly corresponded to the diverged lineages in the phylogenetic trees [67]. In this study, half of the species were identified correctly using this method, while other species were clustered together with their relatives. This corresponded with sections based on morphology to some extent (Supplementary Table S11). 

Recently, the development of methods based on molecular diagnostic characters (MDCs) comes into view; the number of observed MDCs has a particularly large impact on the efficiency of species identification [68]. We conducted an MDC analysis of five datasets (complete cp genomes, five coding genes, six noncoding regions, *ycf1*, and *rpl32*-*trnL^UAG^*); the results revealed that *B*. *crassipes* possessed the most abundant MDCs of the *Bulbophyllum* species, possibly related to the insertion observed in the genome structure (Figure 1 and Figure 6). The number of MDCs of complete cp genomes was remarkable, while three species could not be identified on the basis of differences in the datasets of *rpl32*-*trnL^UAG^* (Figure 6, Supplementary Table S12).

### 3.6. Phylogenetic Analysis

Complete cp genomes are valuable resources for analyzing phylogenetic relationships; they have been extensively used for phylogenetic analysis across different plant groups [24,32]. Our phylogenetic analysis of *Bulbophyllum*, based on complete cp genome and 68 CDSs (Figure 7, Supplementary Figure S5), provided strong support for the monophyly of the Neotropical clade, sects *Lemniscata*, *Racemosae*, and *Macrostylida* (BS ≥ 98, PP = 1.00), in agreement with previous studies [5,27,30,36]. The branch topology and node support rates compared to the phylogenetic relationships constructed using traditional molecular markers also improved [4,5] (Figure 7, Supplementary Figure S5). In addition, *B*. *ambrosia*, previously assigned to sect *Leopardinae*, was distantly related to other two species (*B*. *leopardimum* and *B*. *pectinatum*) [5], a result corroborated here. It was noteworthy that *B*. *hamatum*, being a member of sect *Cirrhopetalum* and closely related to *B*. *omerandrum* based on a morphological comparison [19], was close to two species from sect *Brachyantha* (*B*. *tianguii* and *B*. *violaceolabellum*) with high support (Figure 7, Supplementary Figure S5). Two species, i.e., *B*. *ningboense* and *B*. *gedangense*, were identified as unplaced along the spine of *Bulbophyllum* by Lin et al. and Luo et al. [69,70]. *B*. *ningboense*, a species similar to *B. chrondriophorum* morphologically, was a sister to *B*. *pingnanense* and *B*. *inconspicuum* (Figure 7), with lateral sepals connected partly and sub-umbellate raceme [19], basically in accordance with the characteristics of sect *Cirrhopetalum*. The phylogenetic analysis further strongly supported that *B*. *ningboense* is closely related to *B. pingnanense* within the sect *Cirrhopetalum*. *B*. *gedangense*, morphologically similar to *B. psychoon* and *B. scabractum*, was close to the single species *B*. *ambrosia*. It appears that more sampling and more evidence are required to understand the evolutionary history of *B*. *gedangense*. Our results generally indicated that there was an overlap of species from different sections, especially sects *Brachyantha*, *Cirrhopetalum*, and *Leopardinae*. The conclusions of previous studies found that the boundaries between these sections should be re-evaluated [5,27,71]. However, our phylogenetic analysis showed that species from sect *Ephippium* and sect *Desmosanthes*, as well as sect *Stenochilus* and sect *Reptantia*, respectively, were sister groups, which might due to limited sampling. Therefore, additional cp genomes from *Bulbophyllum* individuals are necessary to further investigate phylogeny, especially at lower taxonomic levels.

## 4. Materials and Methods

### 4.1. Taxon Sampling and DNA Sequencing

In this study, we sequenced seven *Bulbophyllum* species (*B*. *ambrosia*, *B*. *crassipes*, *B*. *farreri*, *B*. *hamatum*, *B*. *shanicum*, *B*. *triste*, and *B*. *violaceolabellum*), and their voucher specimens were stored at the herbarium of the College of Forestry, Fujian Agriculture and Forestry University (FJFC). Total genome DNA was extracted using a modified cetyltrimethylammonium bromide (CTAB) method [72]. Sequencing was carried out at Berry Genomics (Beijing, China) using the Illumina HiSeq 2500 platform, with a read length of 150 bp. Approximately 10 Gb of raw data was obtained for each species. In addition to our newly sequenced data, we downloaded the available chloroplast genomes of 21 other *Bulbophyllum* species from GenBank (Table 1).

### 4.2. Chloroplast Genome Assembly and Annotation

We employed the GetOrganelle pipeline v1.7.5 for de novo cp genome assembly with the default parameters [73]. Subsequently, the “fastg” file was manually examined, and lower-quality fragments were removed using Bandage v.0.8.1 to obtain circular cp genomes [74]. Gene annotation was carried out using the PGA (Plastid Genome Annotator) software (https://github.com/quxiaojian/PGA, accessed on 25 September 2023) [75] with *Bulbophyllum lingii* (MW161052) as the reference genome. Manual checking and adjustments of the annotation results, including the determination of initiation and termination codon positions and the identification of gene pseudogenization or loss, were performed using the Dual Organellar GenoMe Annotator (DOGMA) (https://dogma.ccbb.utexas.edu/, accessed on 25 September 2023) [76] and Geneious v11.0.11 [77]. Further, the circular genome map was generated online using OGDRAW (https://chlorobox.mpimp-golm.mpg.de/OGDraw, accessed on 1 November 2023) [78]. The annotated cp genome sequences were submitted to NCBI (http://www.ncbi.nlm.nih.gov, accessed on 9 February 2024) (Table 1). All cp genomes obtained from NCBI underwent reannotation using the PGA tool. Geneious v11.0.11 was employed to analyze the length and guanine–cytosine (GC) content of the entire chloroplast genome, including the large single copy (LSC), small single copy (SSC), and inverted repeat (IR) regions. Additionally, we examined the number of genes and categories.

### 4.3. Repeat Sequence Characterization

We identified four types of long repeats within the chloroplast genomes of 28 *Bulbophyllum* species using the REPuter program (https://bibiserv.cebitec.uni-bielefeld.de/reputer, accessed on 1 November 2023) [79]. The parameters for repeat identification were set as follows: (1) hamming distance = 3; (2) minimum repeat size ≥ 30 bp; and (3) maximum computed repeats of 50 bp. To determine the positions and types of microsatellites (SSRs), we employed the microsatellite identification tool MISA (https://webblast.ipk-gatersleben.de/misa/, accessed on 1 November 2023) [80]. We used the following thresholds: 10, 5, 4, 3, 3, and 3 for mono-, di-, tri-, tetra-, penta-, and hexa-nucleotides, respectively [30]. The characteristics of repeat sequences were visualized using the R package *ggplot2* [81].

### 4.4. Relative Synonymous Codon Usage Analysis

Codon usage and relative synonymous codon usage (RSCU) values were estimated using Codon W, accessible at http://codonw.sourceforge.net/ (accessed on 1 November 2023) [82]. To minimize sampling errors, we excluded repeat sequences and protein-coding regions (CDSs) shorter than 300 bp from the codon usage calculations. This step was necessary since short CDSs can lead to estimation errors in codon usage. TBtools v1.1047 was employed to create the heat map for the RSCU analysis [83].

### 4.5. Sequence Divergence and Nucleotide Diversity

To investigate variations in the boundaries of the LSC/IR/SSC regions in 28 *Bulbophyllum* chloroplast genomes, we conducted the SC/IR boundary analyses using the Perl script CPJSdraw.pl (https://github.com/xul962464/CPJSdraw, accessed on 1 November 2023). For visualizing identity across the 28 cp genomes, we employed the shuffle-LAGAN mode of the mVISTA program, with *B*. *affine* (LC556091) as the reference genome (http://genome.lbl.gov/vista/mvista/submit.shtml, accessed on 1 November 2023) [84]. Mauve was utilized to perform analyses of cp genome rearrangement using default “seed families” and default values. In all sequences, one of the IR regions was consistently removed [85]. The nucleotide variability (Pi) for the 28 *Bulbophyllum* cp genomes and the 68 protein-coding genes was calculated using DnaSP v6.0, with a window length of 100 bp and a step size of 25 bp [86].

### 4.6. Molecular Diagnostic Characters (MDCs)

The Automatic Barcode Gap Discovery (ABGD) analysis was conducted online (https://bioinfo.mnhn.fr/abi/public/abgd/abgdweb.html, accessed on 9 February 2024) using the Kimura [K80] TS/TV 2.0 model and specific settings (Pmin = 0.001, Pmax = 0.01, Steps = 10, X = 0.1, Nb bins = 20) for primary species delimitation [66]. Five protein-coding genes and six noncoding sequences were selected for species discrimination analysis according to the Pi values. The MDC analysis was performed using the FastaChar v. 0.2.4 software [87]. The analysis was performed by comparing each species with the other members of *Bulbophyllum* included in this study. 

### 4.7. Phylogenetic Analysis

In accordance with previous molecular systematic studies [27,36,40], we selected a total of 33 chloroplast genomes from 33 species for this study. The selection includes 28 species from *Bulbophyllum* and five species from *Dendrobium* (*D*. *chrysanthum*, *D*. *findlayanum*, *D*. *hercoglossum*, *D*. *longicornu*, and *D*. *moschatum*), which serve as the outgroups (Supplementary Table S9). A total of 68 protein-coding genes (excluding *ndh* genes due to their widespread loss or truncation in *Bulbophyllum*) were extracted using PhyloSuite v1.2.2 [88], and we aligned them using MAFFT v.7 [89]. The complete chloroplast genomes were aligned using MAFFT and trimmed using TrimAl v1.2 to remove poorly aligned positions [90]. For the phylogenetic analysis, we utilized the CIPRES Science Gateway, specifically RaxML-HPC2 on XSEDE 8.2.12, PAUP on XSEDE 4.a168, and MrBayes on XSEDE 3.2.7, applying three methods, including maximum likelihood (ML), maximum parsimony (MP), and Bayesian inference (BI) [91]. In the ML analysis, we specified the GTRGAMMA model for all datasets and calculated bootstrap values on 1000 bootstrap replicates using heuristic searches [92,93]. In the MP analysis, we conducted a heuristic search involving 1000 random addition sequence repeats, employing TBR branch switching. All characters were treated as equally weighted and unordered. In the BI analysis, we utilized the GTR + I + Γ substitution model with MrBayes v. 3.2.7 [94]. The Markov chain Monte Carlo (MCMC) algorithm was run for 10,000,000 generations, with one tree sampled every 100 generations. We discarded the first 25% of trees as burn-ins to construct majority-rule consensus trees and estimate posterior probabilities (PPs).

## 5. Conclusions

In this study, we obtained the cp genomes of seven *Bulbophyllum* species (*B*. *ambrosia*, *B*. *crassipes*, *B*. *farreri*, *B*. *hamatum*, *B*. *shanicum*, *B*. *triste*, and *B*. *violaceolabellum*) and compared them with 21 related species to investigate cp genome differences. We found that most cp genomes exhibited high similarity in terms of the genome size, gene content, and gene order, and differences were observed in their *ndh* gene composition. Additionally, long-repeat sequences in the cp genomes of *Bulbophyllum* species were abundant, with an obvious A/T preference. A number of exclusive SSRs, present in some species, are useful molecular markers for species identification and detecting genetic diversity. The RSCU analysis revealed that the codon usage bias in *Bulbophyllum* cp genomes showed similar patterns. Six highly variable regions (*rpl32*-*trnL^UAG^*, *trnT^UGU^*-*trnL^UAA^*, *trnF^GAA^*-*ndhJ*, *rps15*-*ycf1*, *rbcL*-*accD*, and *psbI*-*trnS^GCU^*) and five coding sequences (*ycf1*, *rps12*, *matK*, *psbK*, and *rps15*) were identified as potential DNA markers based on nucleotide diversity. Additionally, the number of MDCs that distinguish a species from the others varied from 149 to 8603 in complete cp genomes. Based on the cp genome sequences, 28 *Bulbophyllum* species can be divided into four clades, sects. *Brachyantha*, *Cirrhopetalum*, and *Leopardinae*, defined by morphology, were non-monophyly. This study further supports the significance of cp genomes in elucidating the phylogeny of *Bulbophyllum*.

## Figures and Tables

**Table 1:** Features of the complete chloroplast genomes of 28 *Bulbophyllum* species.

Species	Specimen Voucher	Accession No.	Size (bp)				Number of Genes (Unique)	Protein-Coding Genes (Unique)	tRNA Genes (Unique)	rRNA Genes (Unique)	*ndh* Genes Loss/Pseudogenization	GC% (Total)
			Total	LSC	SSC	IR						
*B. affine*	-	LC556091	148,230	78,178	17,280	26,386	132 (113)	86 (79)	38 (30)	8 (4)	−/−	37.86
*B. ambrosia* *	MHLi or138	PP315905	158,026	85,821	18,622	26,804	132 (113)	86 (79)	38 (30)	8 (4)	−/−	36.95
*B. andersonii*	Yang202201	LC703293	148,255	78,074	17,449	26,366	132 (113)	86 (79)	38 (30)	8 (4)	−/−	37.83
B. crassipes*	MHLi or153	PP315906	165,812	85,690	18,293	30,927	132 (113)	86 (79)	38 (30)	8 (4)	−/−	37.29
*B. disciflorum*	-	LC498826	148,554	79,001	16,797	26,378	131 (112)	77 (70)	38 (30)	8 (4)	1/8	37.94
*B. exaltatum*	Fiorini 218 (HBCB)	MN604054	150,410	83,335	15,380	25,847	129 (110)	76 (70)	38 (30)	8 (4)	3/7	36.80
*B. farreri* *	MHLi or142	PP315907	157,339	85,560	18,228	26,788	132 (113)	86 (79)	38 (30)	8 (4)	−/−	36.96
*B. gedangense*	Y. Luo et al., 1239	MW161053	158,524	86,200	18,632	26,846	132 (113)	86 (79)	38 (30)	8 (4)	−/−	36.80
*B. granulosum*	Mancinelli 1059 (UPCB)	MN604055	151,112	84,492	15,690	25,465	128 (110)	76 (69)	38 (30)	8 (4)	7/2	36.70
*B. hamatum* *	MHLi or160	PP315908	152,654	84,132	16,881	25,822	128 (113)	80 (79)	38 (30)	8 (4)	4/2	36.95
*B. hirtum*	Yang202105	LC642724	147,382	77,587	17,129	26,333	132 (113)	86 (79)	38 (30)	8 (4)	−/−	37.96
*B. inconspicuum*	PDBK2012-0213	MN200377	149,548	85,760	12,136	25,826	127 (108)	78 (71)	38 (30)	8 (4)	5/3	37.00
*B. kwangtungense*	Yang202107	LC642722	145,092	77,192	15,376	26,262	129 (110)	82 (75)	38 (30)	8 (4)	3/1	37.98
*B. leopardinum*	Yang202102	LC642723	147,514	77,762	16,996	26,378	132 (113)	86 (79)	38 (30)	8 (4)	−/−	38.04
*B. lingii*	Y. Luo et al., 2247	MW161052	156,689	84,607	18,244	26,919	132 (113)	86 (79)	38 (30)	8 (4)	−/−	36.80
*B. menghaiense*	XY Wang & ZF Xu 202,003	MW161050	156,550	84,663	18,105	26,891	131 (112)	85 (78)	38 (30)	8 (4)	−/−	36.70
*B. mentosum*	Fiorini 323 (HBCB)	MN604056	150,217	83,640	13,895	26,341	125 (106)	74 (68)	38 (30)	8 (4)	7/5	36.70
*B. ningboense*	-	MW683325	151,052	86,020	13,348	25,842	128 (109)	80 (73)	38 (30)	8 (4)	4/2	37.00
*B. orientale*	Yang202104	LC642725	147,388	77,392	17,206	26,395	132 (113)	86 (79)	38 (30)	8 (4)	−/−	38.01
*B. pectinatum*	-	LC556092	147,169	77,478	17,529	26,081	132 (113)	86 (79)	38 (30)	8 (4)	−/−	38.01
*B. pentaneurum*	Y. Luo et al., 2252	MW161051	156,182	84,240	18,266	26,838	132 (113)	86 (79)	38 (30)	8 (4)	−/−	36.80
*B. pingnanense*	J.F. Liu 201312	MW822749	151,224	86,017	13,497	25,855	128 (109)	80 (73)	38 (30)	8 (4)	4/2	37.00
*B. plumosum*	Imig 606 (HAC)	MN580547	146,401	83,260	11,089	26,026	125 (106)	74 (68)	38 (30)	8 (4)	7/5	36.60
*B. reptans*	Yang202106	LC642726	146,928	77,088	17,038	26,401	132 (113)	86 (79)	38 (30)	8 (4)	−/−	37.98
*B. shanicum* *	MHLi or148	PP315909	158,009	85,657	18,253	27,062	132 (113)	86 (79)	38 (30)	8 (4)	−/−	36.99
*B. tianguii*	-	MZ983368	151,953	83,780	16,683	25,746	127 (108)	77 (70)	38 (30)	8 (4)	5/4	37.00
*B. triste* *	MHLi or145	PP315910	157,429	87,177	18,199	27,039	132 (113)	86 (79)	38 (30)	8 (4)	−/−	37.04
*B. violaceolabellum* *	MHLi or152	PP315911	157,811	85,751	18,445	26,820	132 (113)	86 (79)	38 (30)	8 (4)	−/−	36.87

Note: An asterisk (*) indicates the sequences are newly sequenced in this study, “-” indicated the missing data.

## Data Availability

All the data are provided within this manuscript and Supplementary Materials.

## Supplementary Materials

The supporting information can be downloaded at: https://www.mdpi.com/article/10.3390/ijms25052665/s1.
