# Supplementary material for: The Complete Chloroplast Genomes of Bulbophyllum (Orchidaceae) Species: Insight into Genome Structure Divergence and Phylogenetic Analysis
Source: Int J Mol Sci. 2024 Feb 25;25(5):2665. doi: 10.3390/ijms25052665 (PMC10932260; doi:10.3390/ijms25052665)
Supplement: Supplementary file 1 [file ijms-25-02665-s001.zip › Supplementary Figures.pdf]

Supplementary Figures

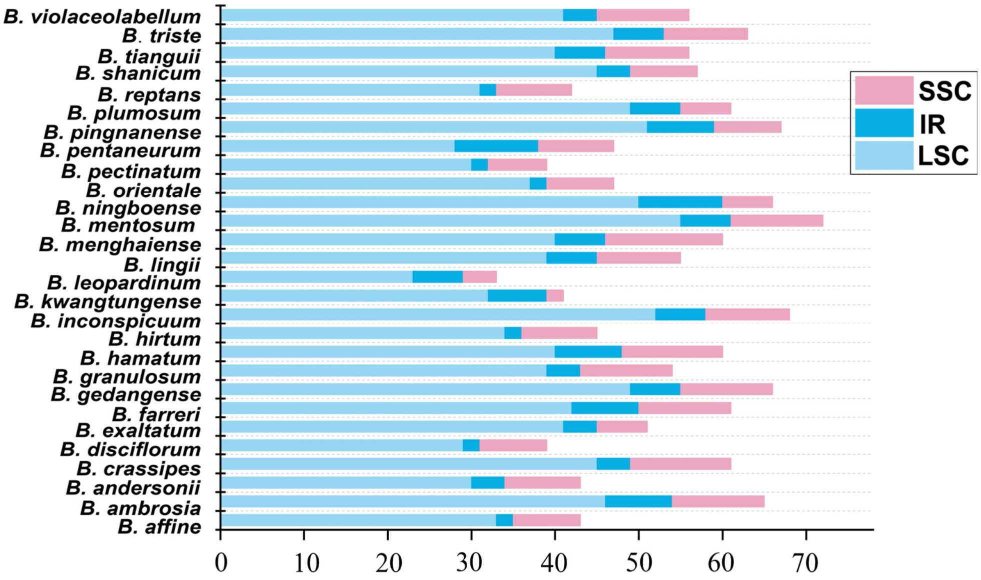

Supplementary Figure S1 Number of SSRs for each *Bulbophyllum* species by location in IR, LSC, and SSC.

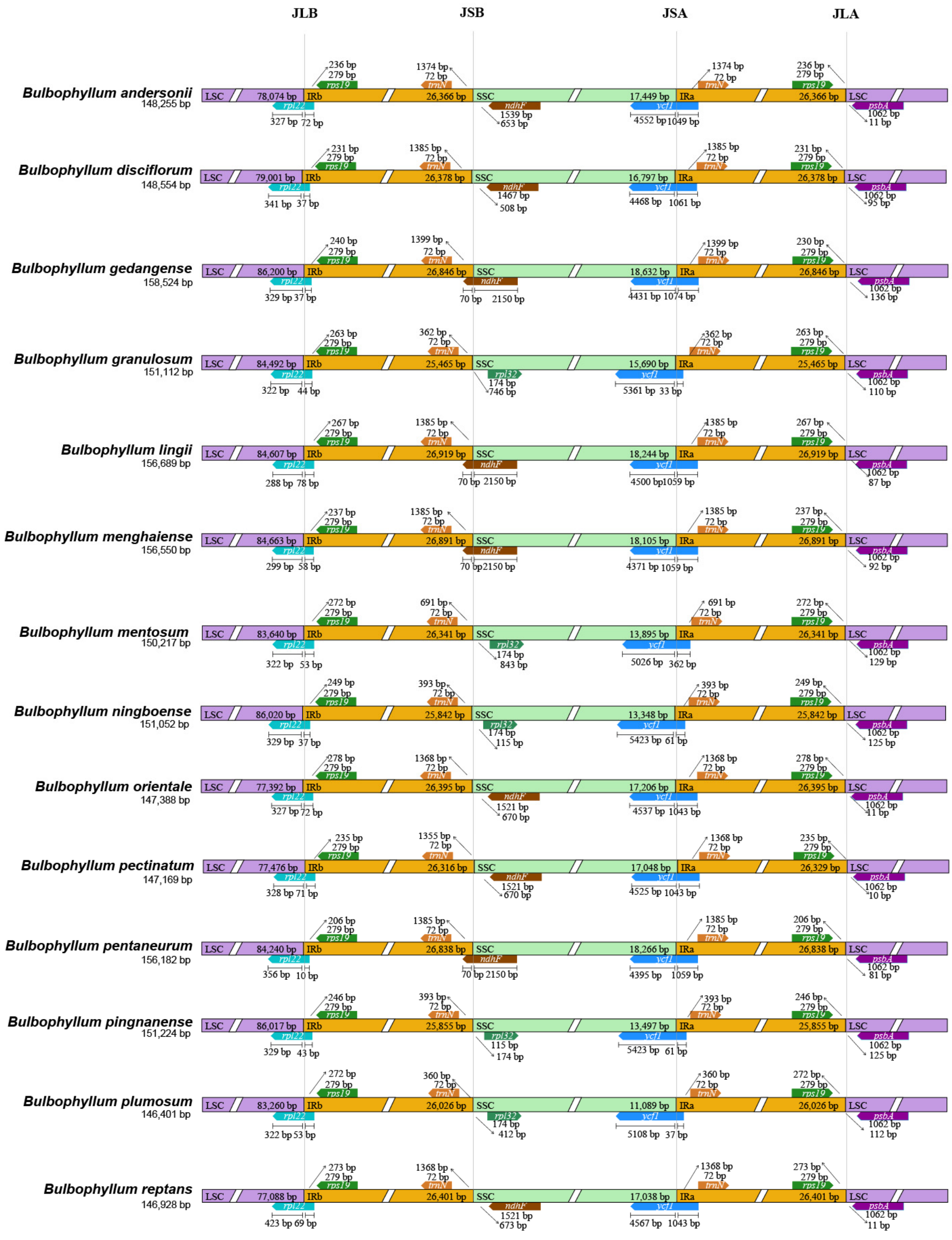

Supplementary Figure S2 Comparison of junctions between the LSC, SSC, and IR regions among the other 14 *Bulbophyllum* cp genomes.

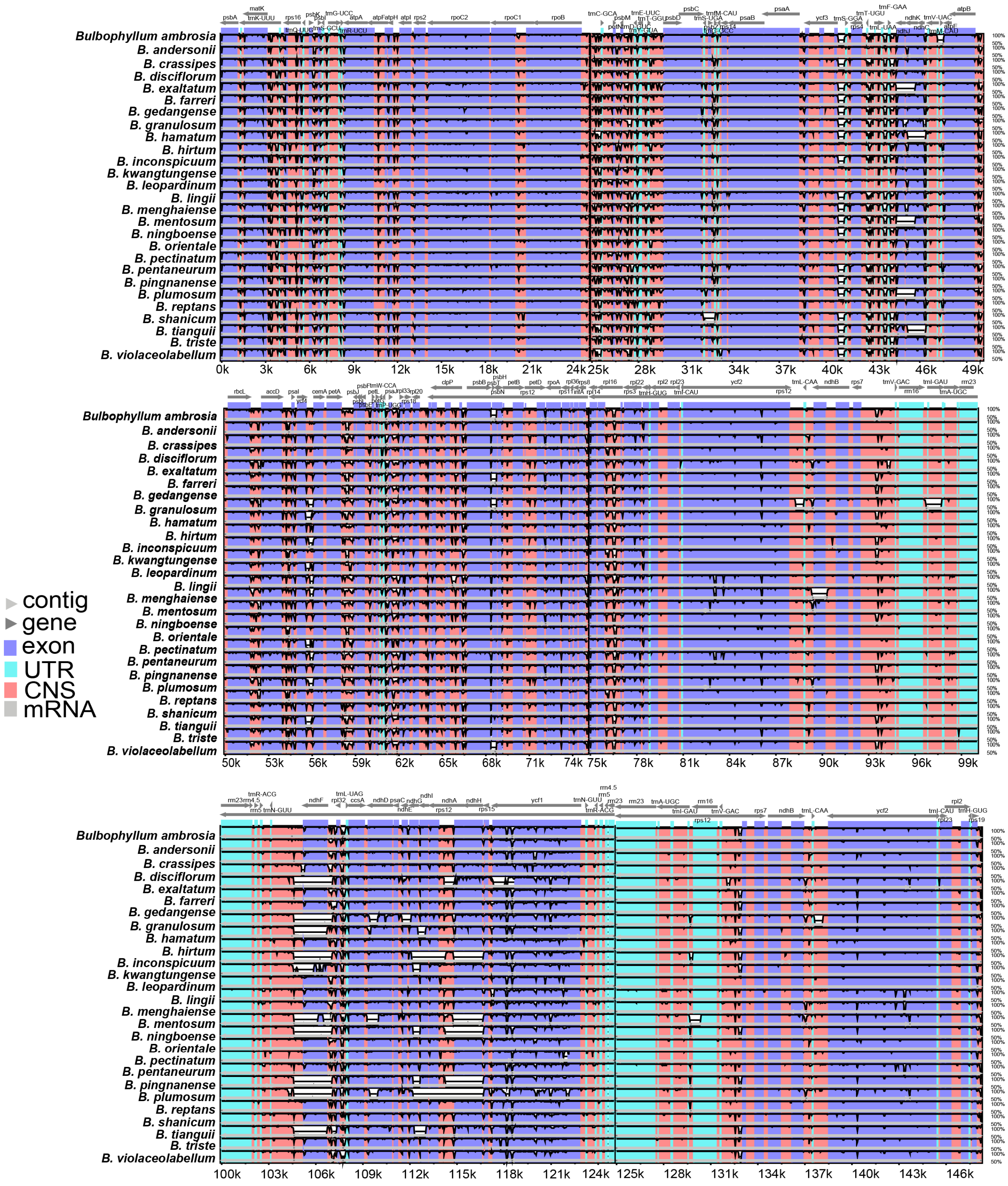

Supplementary Figures S3 Global alignment of 28 *Bulbophyllum* cp genomes using mVISTA with *B. affine* as reference. Thick, gray arrows above the alignment indicate the orientation and position of each gene. The Y-axis represents the identity percentage, ranging from 50 to 100%

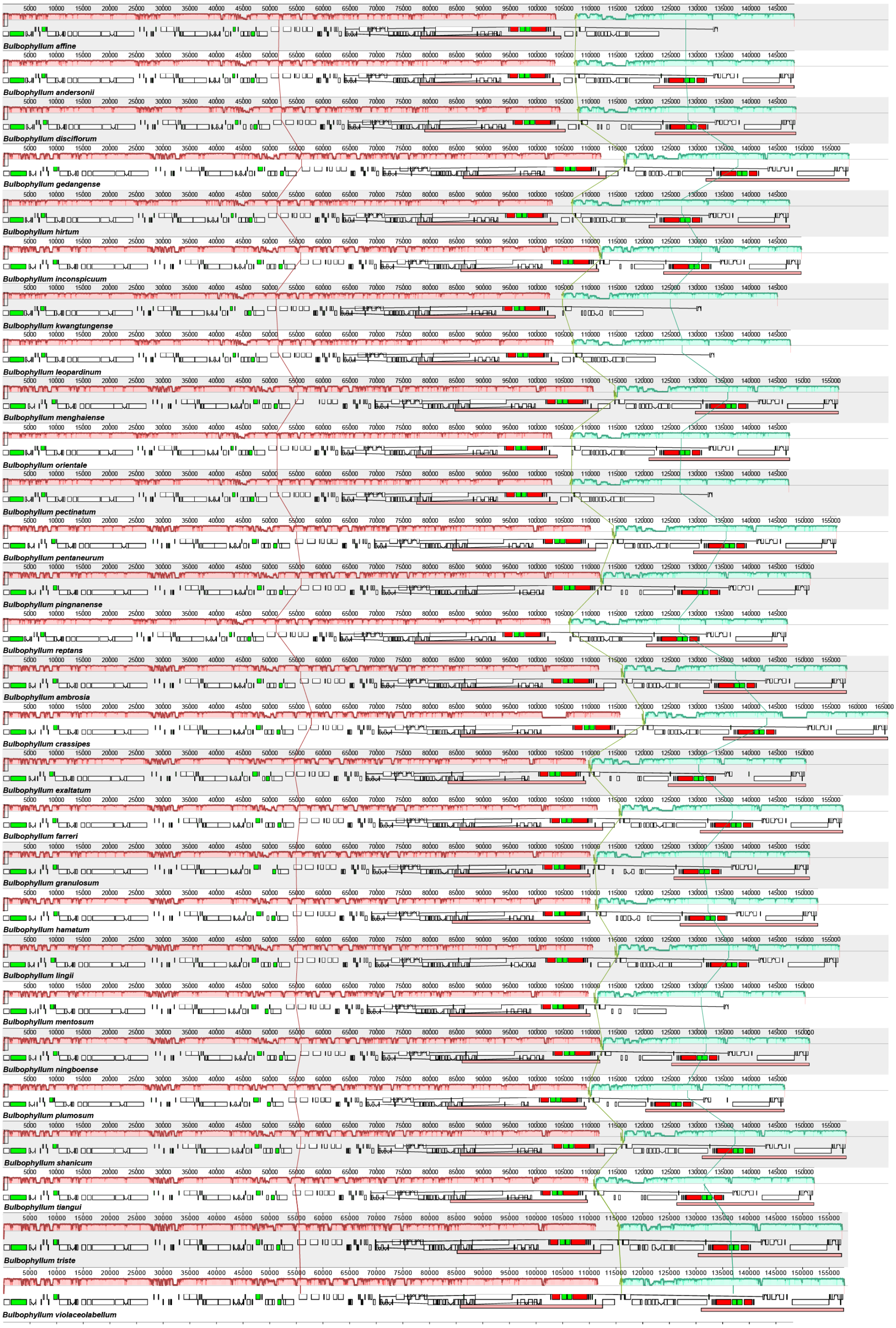

Supplementary Figure S4 Alignment of the 28 *Bulbophyllum* cp genomes (mauve graphs). Local collinear blocks within each alignment are represented by blocks of the same color connected with lines.

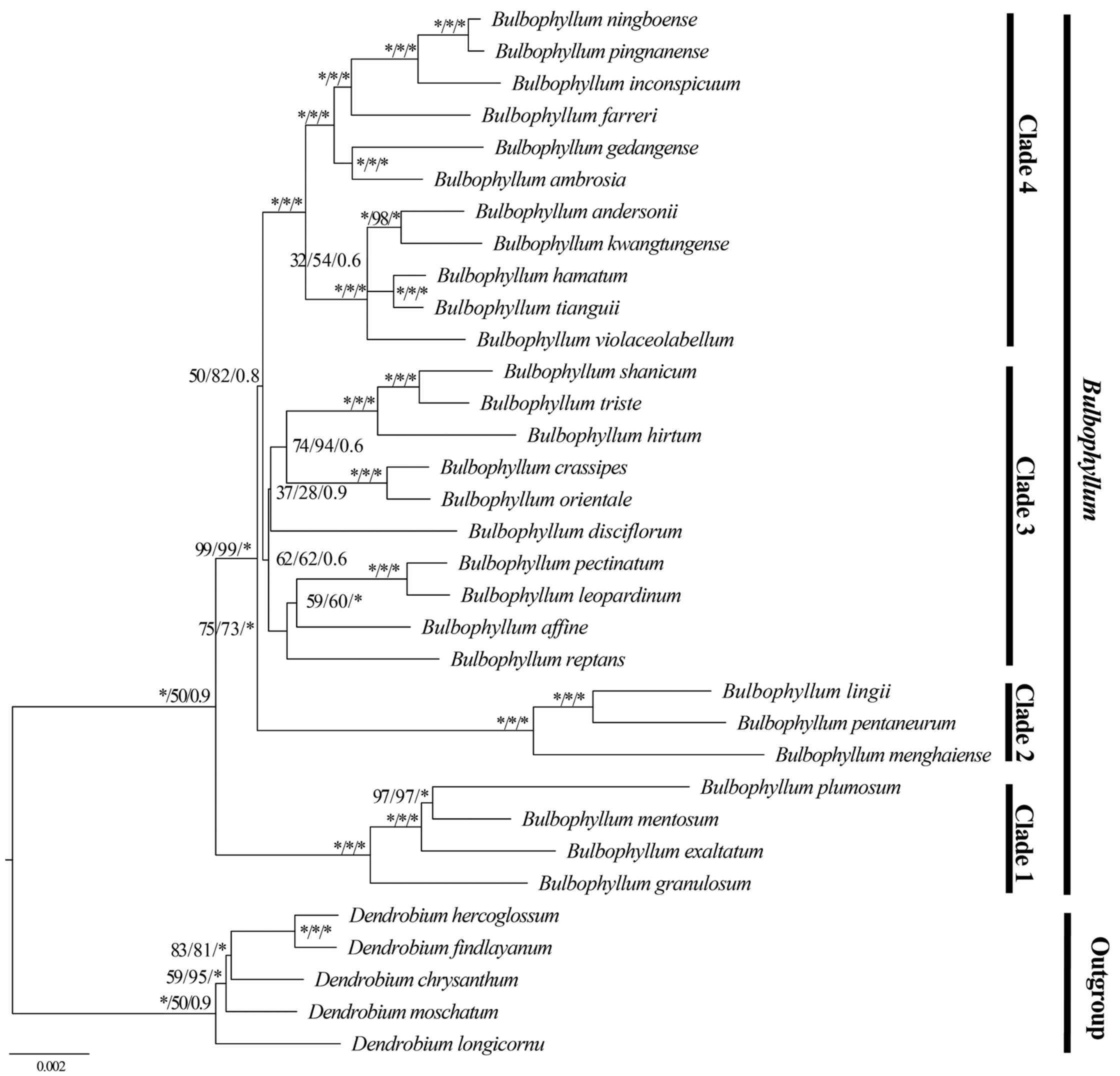

Supplementary Figure S5 The phylogenetic tree of 28 *Bulbophyllum* species and five outgroups based on concatenated 68 protein-coding genes. The numbers near the nodes are bootstrap percentages and Bayesian posterior probabilities (BP_{ML}, BP_{MP}, PP), *node is 100 bootstrap percentage or 1.00 posterior probability.
